# Supplementary material for: Tibolone Pre-Treatment Ameliorates the Dysregulation of Protein Translation and Transport Generated by Palmitic Acid-Induced Lipotoxicity in Human Astrocytes: A Label-Free MS-Based Proteomics and Network Analysis
Source: Int J Mol Sci. 2022 Jun 9;23(12):6454. doi: 10.3390/ijms23126454 (PMC9223656; doi:10.3390/ijms23126454)
Supplement: Supplementary file 1 [file ijms-23-06454-s001.zip › ijms-1726735-supplementary.pdf]

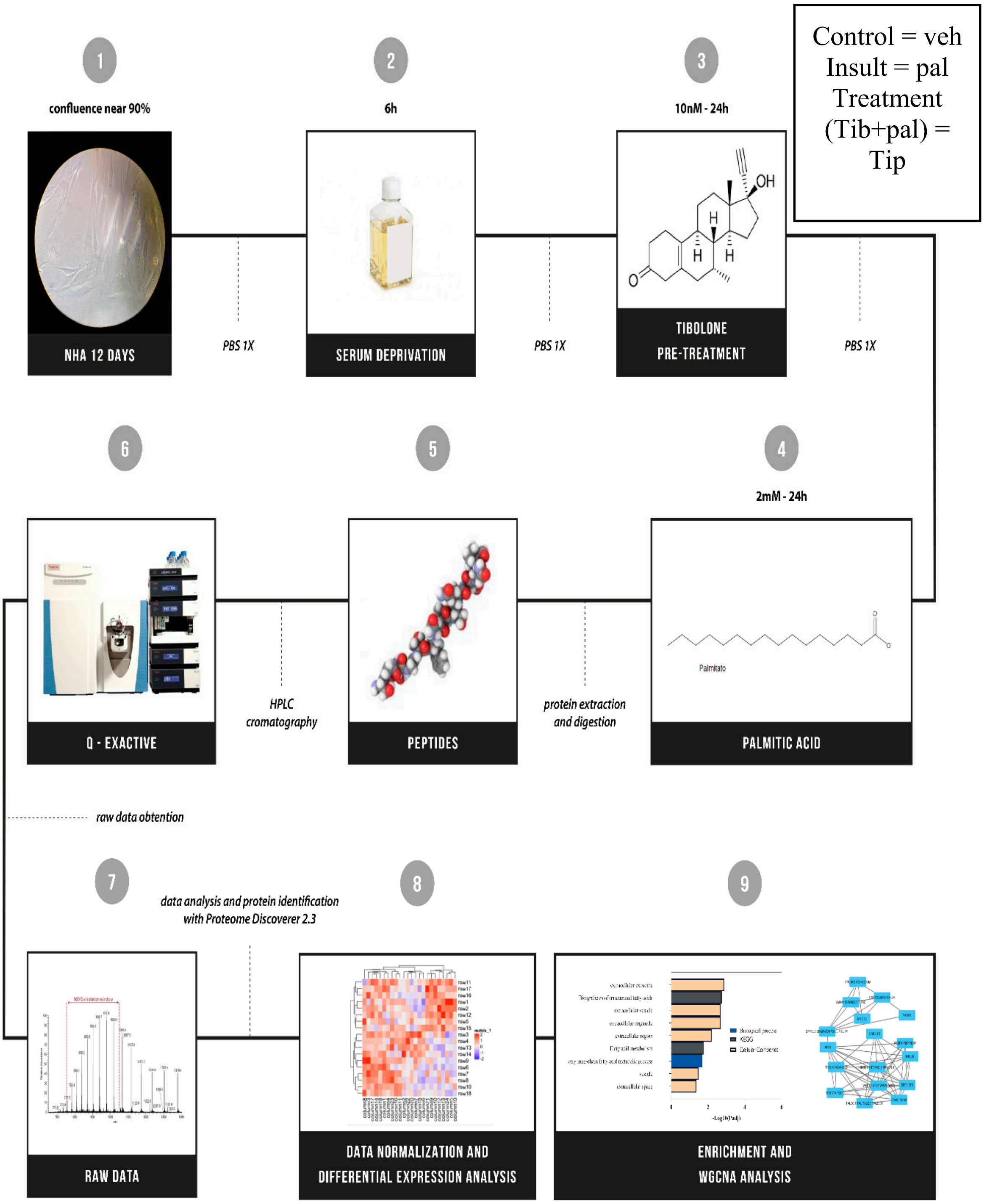

Figure S1. Graphic summary of the methodology used in the study. Normal human astrocytes (NHA) were cultured in ABM until 80% confluency (1), serum-deprived for 6 h (2), and submitted or not to tibolone pre-treatment (3) and palmitic acid (4). Then, sample proteins were extracted, digested (5), and LC-MS/MS-analyzed (6). The obtained raw data (7) were normalized and analyzed (8), and enrichment and WGCNA analyses were performed (9).

Table S1. Full list of differentially expressed proteins in the comparison between pal vs. veh, p < 0.01 and FDR < 0.1.

Entry	Protein names	Gene names	Protein expression
P31949	Protein S100-A11 (Calgizzarin) (Metastatic lymph node gene 70 protein) (MLN 70) (Protein S100-C) (S100 calcium-binding protein A11) [Cleaved into: Protein S100-A11, N-terminally processed]	S100A11 MLN70 S100C	Up-regulated
Q9P2R7	Succinate--CoA ligase [ADP-forming] subunit beta, mitochondrial (EC 6.2.1.5) (ATP-specific succinyl-CoA synthetase subunit beta) (A-SCS) (Succinyl-CoA synthetase beta-A chain) (SCS-betaA)	SUCLA2	Up-regulated
Q12904	Aminoacyl tRNA synthase complex-interacting multifunctional protein 1 (Multisynthase complex auxiliary component p43) [Cleaved into: Endothelial monocyte-activating polypeptide 2 (EMAP-2) (Endothelial monocyte-activating polypeptide II) (EMAP-II) (Small inducible cytokine subfamily E member 1)]	AIMP1 EMAP2 SCYE1	Up-regulated
Q9Y265	RuvB-like 1 (EC 3.6.4.12) (49 kDa TATA box-binding protein-interacting protein) (49 kDa TBP-interacting protein) (54 kDa erythrocyte cytosolic protein) (ECP-54) (INO80 complex subunit H) (Nuclear matrix protein 238) (NMP 238) (Pontin 52) (TIP49a) (TIP60-associated protein 54-alpha) (TAP54-alpha)	RUVBL1 INO80H NMP238 TIP49 TIP49A	Up-regulated
P61204	ADP-ribosylation factor 3	ARF3	Up-regulated
P60484	Phosphatidylinositol 3,4,5-trisphosphate 3-phosphatase and dual-specificity protein phosphatase PTEN (EC 3.1.3.16) (EC 3.1.3.48) (EC 3.1.3.67) (Mutated in multiple advanced cancers 1) (Phosphatase and tensin homolog)	PTEN MMAC1 TEP1	Up-regulated
Q8NGA1	Olfactory receptor 1M1 (Olfactory receptor 19-6) (OR19-6) (Olfactory receptor OR19-5)	OR1M1	Up-regulated
P81605	Dermcidin (EC 3.4.-.-) (Preproteolysin) [Cleaved into: Survival-promoting peptide; DCD-1]	DCD AIDD DSEP	Up-regulated
P13473	Lysosome-associated membrane glycoprotein 2 (LAMP-2) (Lysosome-associated membrane protein 2) (CD107 antigen-like family member B) (LGP-96) (CD antigen CD107b)	LAMP2	Up-regulated
P00403	Cytochrome c oxidase subunit 2 (EC 7.1.1.9) (Cytochrome c oxidase polypeptide II)	MT-CO2 COII COX2 COXII MTCO2	Up-regulated
Q9BW60	Elongation of very long chain fatty acids protein 1 (EC 2.3.1.199) (3-keto acyl-CoA synthase ELOVL1) (ELOVL fatty acid elongase 1) (ELOVL FA elongase 1) (Very long chain 3-ketoacyl-CoA synthase 1) (Very long chain 3-oxoacyl-CoA synthase 1)	ELOVL1 SSC1 CGI-88	Up-regulated
Q9UBI6	Guanine nucleotide-binding protein G(I)/G(S)/G(O) subunit gamma-12	GNG12	Up-regulated
Q6P2Q9	Pre-mRNA-processing-splicing factor 8 (220 kDa U5 snRNP-specific protein) (PRP8 homolog) (Splicing factor Prp8) (p220)	PRPF8 PRPC8	Up-regulated
P62851	40S ribosomal protein S25 (Small ribosomal subunit protein eS25)	RPS25	Up-regulated
P46109	Crk-like protein	CRKL	Up-regulated
P49821	NADH dehydrogenase [ubiquinone] flavoprotein 1, mitochondrial (EC 7.1.1.2) (Complex I-51kD) (CI-	NDUFV1 UQOR1	Up-regulated

	51kD) (NADH dehydrogenase flavoprotein 1) (NADH-ubiquinone oxidoreductase 51 kDa subunit)		
O14737	Programmed cell death protein 5 (TF-1 cell apoptosis-related protein 19) (Protein TFAR19)	PDCD5 TFAR19	Up-regulated
Q9NZ01	Very-long-chain enoyl-CoA reductase (EC 1.3.1.93) (Synaptic glycoprotein SC2) (Trans-2,3-enoyl-CoA reductase) (TER)	TECR GPSN2 SC2	Up-regulated
Q96DG6	Carboxymethylenebutenolidase homolog (EC 3.1.-.-)	CMBL	Up-regulated
Q92888	Rho guanine nucleotide exchange factor 1 (115 kDa guanine nucleotide exchange factor) (p115-RhoGEF) (p115RhoGEF) (Sub1.5)	ARHGEF1	Up-regulated
Q9UJU6	Drebrin-like protein (Cervical SH3P7) (Cervical mucin-associated protein) (Drebrin-F) (HPK1-interacting protein of 55 kDa) (HIP-55) (SH3 domain-containing protein 7)	DBNL CMAP SH3P7 PP5423	Up-regulated
P09913	Interferon-induced protein with tetratricopeptide repeats 2 (IFIT-2) (ISG-54 K) (Interferon-induced 54 kDa protein) (IFI-54K) (P54)	IFIT2 CIG-42 G10P2 IFI54 ISG54	Up-regulated
P09110	3-ketoacyl-CoA thiolase, peroxisomal (EC 2.3.1.16) (Acetyl-CoA acyltransferase) (Beta-ketothiolase) (Peroxisomal 3-oxoacyl-CoA thiolase)	ACAA1 ACAA PTHIO	Up-regulated
P13010	X-ray repair cross-complementing protein 5 (EC 3.6.4.-) (86 kDa subunit of Ku antigen) (ATP-dependent DNA helicase 2 subunit 2) (ATP-dependent DNA helicase II 80 kDa subunit) (CTC box-binding factor 85 kDa subunit) (CTC85) (CTCBF) (DNA repair protein XRCC5) (Ku80) (Ku86) (Lupus Ku autoantigen protein p86) (Nuclear factor IV) (Thyroid-lupus autoantigen) (TLAA) (X-ray repair complementing defective repair in Chinese hamster cells 5 (double-strand-break rejoining))	XRCC5 G22P2	Up-regulated
P12955	Xaa-Pro dipeptidase (X-Pro dipeptidase) (EC 3.4.13.9) (Imidodipeptidase) (Peptidase D) (Proline dipeptidase) (Prolidase)	PEPD PRD	Up-regulated
Q96Q42	Alsin (Amyotrophic lateral sclerosis 2 chromosomal region candidate gene 6 protein) (Amyotrophic lateral sclerosis 2 protein)	ALS2 ALS2CR6 KIAA1563	Down-regulated
P78344	Eukaryotic translation initiation factor 4 gamma 2 (eIF-4-gamma 2) (eIF-4G 2) (eIF4G 2) (Death-associated protein 5) (DAP-5) (p97)	EIF4G2 DAP5 OK/SW-cl.75	Down-regulated
Q9UNE7	E3 ubiquitin-protein ligase CHIP (EC 2.3.2.27) (Antigen NY-CO-7) (CLL-associated antigen KW-8) (Carboxy terminus of Hsp70-interacting protein) (RING-type E3 ubiquitin transferase CHIP) (STIP1 homology and U box-containing protein 1)	STUB1 CHIP PP1131	Down-regulated
P55263	Adenosine kinase (AK) (EC 2.7.1.20) (Adenosine 5'-phosphotransferase)	ADK	Down-regulated
Q16204	Coiled-coil domain-containing protein 6 (Papillary thyroid carcinoma-encoded protein) (Protein H4)	CCDC6 D10S170 TST1	Down-regulated
P48047	ATP synthase subunit O, mitochondrial (ATP synthase peripheral stalk subunit OSCP) (Oligomycin sensitivity conferral protein) (OSCP)	ATP5PO ATP5O ATPO	Down-regulated
Q9Y333	U6 snRNA-associated Sm-like protein LSm2 (Protein G7b) (Small nuclear ribonuclear protein D homolog) (snRNP core Sm-like protein Sm-x5)	LSM2 C6orf28 G7B	Down-regulated
Q13561	Dynactin subunit 2 (50 kDa dynein-associated polypeptide) (Dynactin complex 50 kDa subunit) (DCTN-50) (p50 dynamitin)	DCTN2 DCTN50	Down-regulated

Q9P2B4	CTTNBP2 N-terminal-like protein	CTTNBP2NL KIAA1433	Down-regulated
Q15404	Ras suppressor protein 1 (RSP-1) (Rsu-1)	RSU1 RSP1	Down-regulated
O60884	DnaJ homolog subfamily A member 2 (Cell cycle progression restoration gene 3 protein) (Dnj3) (Dj3) (HIRA-interacting protein 4) (Renal carcinoma antigen NY-REN-14)	DNAJA2 CPR3 HIRIP4	Down-regulated
P16035	Metalloproteinase inhibitor 2 (CSC-21K) (Tissue inhibitor of metalloproteinases 2) (TIMP-2)	TIMP2	Down-regulated
Q9NPA8	Transcription and mRNA export factor ENY2 (Enhancer of yellow 2 transcription factor homolog)	ENY2 DC6	Down-regulated
P61927	60S ribosomal protein L37 (G1.16) (Large ribosomal subunit protein eL37)	RPL37	Down-regulated
O95373	Importin-7 (Imp7) (Ran-binding protein 7) (RanBP7)	IPO7 RANBP7	Down-regulated
Q92890	Ubiquitin recognition factor in ER-associated degradation protein 1 (Ubiquitin fusion degradation protein 1) (UB fusion protein 1)	UFD1 UFD1L	Down-regulated
P84090	Enhancer of rudimentary homolog	ERH	Down-regulated
P12270	Nucleoprotein TPR (Megator) (NPC-associated intranuclear protein) (Translocated promoter region protein)	TPR	Down-regulated
O00571	ATP-dependent RNA helicase DDX3X (EC 3.6.4.13) (CAP-Rf) (DEAD box protein 3, X-chromosomal) (DEAD box, X isoform) (DBX) (Helicase-like protein 2) (HLP2)	DDX3X DBX DDX3	Down-regulated
O43795	Unconventional myosin-Ib (MYH-1c) (Myosin I alpha) (MMI-alpha) (MMIa)	MYO1B	Down-regulated
P61313	60S ribosomal protein L15 (Large ribosomal subunit protein eL15)	RPL15 EC45 TCBAP0781	Down-regulated
Q14677	Clathrin interactor 1 (Clathrin-interacting protein localized in the trans-Golgi region) (Clint) (Enthoprotin) (Epsin-4) (Epsin-related protein) (EpsinR)	CLINT1 ENTH EPN4 EPNR KIAA0171	Down-regulated
Q6NYC8	Phostensin (Protein phosphatase 1 F-actin cytoskeleton-targeting subunit) (Protein phosphatase 1 regulatory subunit 18)	PPP1R18 HKMT1098 KIAA1949	Down-regulated
P05198	Eukaryotic translation initiation factor 2 subunit 1 (Eukaryotic translation initiation factor 2 subunit alpha) (eIF-2-alpha) (eIF-2A) (eIF-2alpha)	EIF2S1 EIF2A	Down-regulated
Q13409	Cytoplasmic dynein 1 intermediate chain 2 (Cytoplasmic dynein intermediate chain 2) (Dynein intermediate chain 2, cytosolic) (DH IC-2)	DYNC1I2 DNCI2 DNCIC2	Down-regulated
Q9UHB9	Signal recognition particle subunit SRP68 (SRP68) (Signal recognition particle 68 kDa protein)	SRP68	Down-regulated
Q9UHV9	Prefoldin subunit 2	PFDN2 PFD2 HSPC231	Down-regulated
P53621	Coatomer subunit alpha (Alpha-coat protein) (Alpha-COP) (HEP-COP) (HEPCOP) [Cleaved into: Xenin (Xenopsin-related peptide); Proxenin]	COPA	Down-regulated
P53618	Coatomer subunit beta (Beta-coat protein) (Beta-COP)	COPB1 COPB MSTP026	Down-regulated

Table S2. Full list of differentially expressed proteins in tip vs. veh, $p < 0.01$ and FDR < 0.1.

Entry	Protein names	Gene names	Protein expression
Q16658	Fascin (55 kDa actin-bundling protein) (Singed-like protein) (p55)	FSCN1 FAN1 HSN SNL	Up-regulated
Q9Y3D6	Mitochondrial fission 1 protein (FIS1 homolog) (hFis1) (Tetratricopeptide repeat protein 11) (TPR repeat protein 11)	FIS1 TTC11 CGI-135	Up-regulated
Q9NQC3	Reticulon-4 (Foocen) (Neurite outgrowth inhibitor) (Nogo protein) (Neuroendocrine-specific protein) (NSP) (Neuroendocrine-specific protein C homolog) (RTN-x) (Reticulon-5)	RTN4 KIAA0886 NOGO My043 SP1507	Up-regulated
O95819	Mitogen-activated protein kinase kinase kinase kinase 4 (EC 2.7.11.1) (HPK/GCK-like kinase HGK) (MAPK/ERK kinase kinase kinase 4) (MEK kinase kinase 4) (MEKKK 4) (Nck-interacting kinase)	MAP4K4 HGK KIAA0687 NIK	Up-regulated
Q4KWH8	1-phosphatidylinositol 4,5-bisphosphate phosphodiesterase eta-1 (EC 3.1.4.11) (Phosphoinositide phospholipase C-eta-1) (Phospholipase C-eta-1) (PLC-eta-1) (Phospholipase C-like protein 3) (PLC-L3)	PLCH1 KIAA1069 PLCL3	Up-regulated
P63167	Dynein light chain 1, cytoplasmic (8 kDa dynein light chain) (DLC8) (Dynein light chain LC8-type 1) (Protein inhibitor of neuronal nitric oxide synthase) (PIN)	DYNLL1 DLC1 DNCL1 DNCLC1 HDLC1	Up-regulated
O75439	Mitochondrial-processing peptidase subunit beta (EC 3.4.24.64) (Beta-MPP) (P-52)	PMPCB MPPB	Up-regulated
P05141	ADP/ATP translocase 2 (ADP,ATP carrier protein 2) (ADP,ATP carrier protein, fibroblast isoform) (Adenine nucleotide translocator 2) (ANT 2) (Solute carrier family 25 member 5) [Cleaved into: ADP/ATP translocase 2, N-terminally processed]	SLC25A5 ANT2	Up-regulated
Q9UHB9	Signal recognition particle subunit SRP68 (SRP68) (Signal recognition particle 68 kDa protein)	SRP68	Up-regulated
O75165	DnaJ homolog subfamily C member 13 (Required for receptor-mediated endocytosis 8) (RME-8)	DNAJC13 KIAA0678 RME8	Up-regulated
O43169	Cytochrome b5 type B (Cytochrome b5 outer mitochondrial membrane isoform)	CYB5B CYB5M OMB5	Up-regulated
P58546	Myotrophin (Protein V-1)	MTPN	Up-regulated
P63096	Guanine nucleotide-binding protein G(i) subunit alpha-1 (Adenylate cyclase-inhibiting G alpha protein)	GNAI1	Up-regulated
Q12904	Aminoacyl tRNA synthase complex-interacting multifunctional protein 1 (Multisynthase complex auxiliary	AIMP1 EMAP2 SCYE1	Up-regulated

	component p43) [Cleaved into: Endothelial monocyte-activating polypeptide 2 (EMAP-2) (Endothelial monocyte-activating polypeptide II) (EMAP-II) (Small inducible cytokine subfamily E member 1)]		
Q13162	Peroxiredoxin-4 (EC 1.11.1.24) (Antioxidant enzyme AOE372) (AOE37-2) (Peroxiredoxin IV) (Prx-IV) (Thioredoxin peroxidase AO372) (Thioredoxin-dependent peroxide reductase A0372) (Thioredoxin-dependent peroxiredoxin 4)	PRDX4	Up-regulated
P21589	5'-nucleotidase (5'-NT) (EC 3.1.3.5) (Ecto-5'-nucleotidase) (CD antigen CD73)	NT5E NT5 NTE	Up-regulated
O75340	Programmed cell death protein 6 (Apoptosis-linked gene 2 protein homolog) (ALG-2)	PDCD6 ALG2	Up-regulated
P09914	Interferon-induced protein with tetratricopeptide repeats 1 (IFIT-1) (Interferon-induced 56 kDa protein) (IFI-56K) (P56)	IFIT1 G10P1 IFI56 IFNAI1 ISG56	Up-regulated
Q14108	Lysosome membrane protein 2 (85 kDa lysosomal membrane sialoglycoprotein) (LGP85) (CD36 antigen-like 2) (Lysosome membrane protein II) (LIMP II) (Scavenger receptor class B member 2) (CD antigen CD36)	SCARB2 CD36L2 LIMP2 LIMPII	Up-regulated
P47755	F-actin-capping protein subunit alpha-2 (CapZ alpha-2)	CAPZA2	Up-regulated
Q6IBS0	Twinfilin-2 (A6-related protein) (hA6RP) (Protein tyrosine kinase 9-like) (Twinfilin-1-like protein)	TWF2 PTK9L MSTP011	Up-regulated
P14174	Macrophage migration inhibitory factor (MIF) (EC 5.3.2.1) (Glycosylation-inhibiting factor) (GIF) (L-dopachrome isomerase) (L-dopachrome tautomerase) (EC 5.3.3.12) (Phenylpyruvate tautomerase)	MIF GLIF MMIF	Up-regulated
P60903	Protein S100-A10 (Calpactin I light chain) (Calpactin-1 light chain) (Cellular ligand of annexin II) (S100 calcium-binding protein A10) (p10 protein) (p11)	S100A10 ANX2LG CAL1L CLP11	Up-regulated
P06703	Protein S100-A6 (Calcyclin) (Growth factor-inducible protein 2A9) (MLN 4) (Prolactin receptor-associated protein) (PRA) (S100 calcium-binding protein A6)	S100A6 CACY	Up-regulated
P06744	Glucose-6-phosphate isomerase (GPI) (EC 5.3.1.9) (Autocrine motility factor) (AMF) (Neuroleukin) (NLK) (Phosphoglucose isomerase) (PGI)	GPI	Up-regulated

	(Phosphohexose isomerase) (PHI) (Sperm antigen 36) (SA-36)		
P52594	Arf-GAP domain and FG repeat-containing protein 1 (HIV-1 Rev-binding protein) (Nucleoporin-like protein RIP) (Rev-interacting protein) (Rev/Rex activation domain-binding protein)	AGFG1 HRB RAB RIP	Up-regulated
Q03518	Antigen peptide transporter 1 (APT1) (ATP-binding cassette sub-family B member 2) (Peptide supply factor 1) (Peptide transporter PSF1) (PSF-1) (Peptide transporter TAP1) (Peptide transporter involved in antigen processing 1) (Really interesting new gene 4 protein)	TAP1 ABCB2 PSF1 RING4 Y3	Up-regulated
P07355	Annexin A2 (Annexin II) (Annexin-2) (Calpactin I heavy chain) (Calpactin-1 heavy chain) (Chromobindin-8) (Lipocortin II) (Placental anticoagulant protein IV) (PAP-IV) (Protein I) (p36)	ANXA2 ANX2 ANX2L4 CAL1H LPC2D	Up-regulated
Q92882	Osteoclast-stimulating factor 1	OSTF1	Up-regulated
P62851	40S ribosomal protein S25 (Small ribosomal subunit protein eS25)	RPS25	Up-regulated
O60502	Protein O-GlcNAcase (OGA) (EC 3.2.1.169) (Beta-N-acetylglucosaminidase) (Beta-N-acetylhexosaminidase) (Beta-hexosaminidase) (Meningioma-expressed antigen 5) (N-acetyl-beta-D-glucosaminidase) (N-acetyl-beta-glucosaminidase) (Nuclear cytoplasmic O-GlcNAcase and acetyltransferase) (NCOAT)	OGA HEXC KIAA0679 MEA5 MGEA5	Up-regulated
Q9HAY6	Beta,beta-carotene 15,15'-dioxygenase (EC 1.13.11.63) (Beta-carotene dioxygenase 1) (Beta-carotene oxygenase 1)	BCO1 BCDO BCDO1 BCMO1	Up-regulated
P60484	Phosphatidylinositol 3,4,5-trisphosphate 3-phosphatase and dual-specificity protein phosphatase PTEN (EC 3.1.3.16) (EC 3.1.3.48) (EC 3.1.3.67) (Mutated in multiple advanced cancers 1) (Phosphatase and tensin homolog)	PTEN MMAC1 TEP1	Up-regulated
Q96TA1	Protein Niban 2 (Meg-3) (Melanoma invasion by ERK) (MINERVA) (Niban-like protein 1) (Protein FAM129B)	NIBAN2 C9orf88 FAM129B	Up-regulated
Q92888	Rho guanine nucleotide exchange factor 1 (115 kDa guanine nucleotide exchange factor) (p115-RhoGEF) (p115RhoGEF) (Sub1.5)	ARHGEF1	Up-regulated
P84085	ADP-ribosylation factor 5	ARF5	Up-regulated
P04264	Keratin, type II cytoskeletal 1 (67 kDa cytokeratin) (Cytokeratin-1) (CK-1) (Hair alpha protein)	KRT1 KRTA	Up-regulated

	(Keratin-1) (K1) (Type-II keratin Kb1)		
P49902	Cytosolic purine 5'-nucleotidase (EC 3.1.3.5) (Cytosolic 5'-nucleotidase II)	NT5C2 NT5B NT5CP PNT5	Up-regulated
Q9Y646	Carboxypeptidase Q (EC 3.4.17.-) (Lysosomal dipeptidase) (Plasma glutamate carboxypeptidase)	CPQ LCH1 PGCP	Up-regulated
P29692	Elongation factor 1-delta (EF-1-delta) (Antigen NY-CO-4)	EEF1D EF1D	Up-regulated
Q8IWB7	WD repeat and FYVE domain-containing protein 1 (FYVE domain-containing protein localized to endosomes 1) (FENS-1) (Phosphoinositide-binding protein 1) (WD40- and FYVE domain-containing protein 1) (Zinc finger FYVE domain-containing protein 17)	WDFY1 FENS1 KIAA1435 WDF1 ZFYVE17	Up-regulated
Q96DG6	Carboxymethylenebutenolidase homolog (EC 3.1.-.-)	CMBL	Up-regulated
Q5QNW6	Histone H2B type 2-F (H2B-clustered histone 18)	H2BC18 HIST2H2BF	Up-regulated
Q7L014	Probable ATP-dependent RNA helicase DDX46 (EC 3.6.4.13) (DEAD box protein 46) (PRP5 homolog)	DDX46 KIAA0801	Up-regulated
Q9BW60	Elongation of very long chain fatty acids protein 1 (EC 2.3.1.199) (3-keto acyl-CoA synthase ELOVL1) (ELOVL fatty acid elongase 1) (ELOVL FA elongase 1) (Very long chain 3-ketoacyl-CoA synthase 1) (Very long chain 3-oxoacyl-CoA synthase 1)	ELOVL1 SSC1 CGI-88	Up-regulated
O14879	Interferon-induced protein with tetratricopeptide repeats 3 (IFIT-3) (CIG49) (ISG-60) (Interferon-induced 60 kDa protein) (IFI-60K) (Interferon-induced protein with tetratricopeptide repeats 4) (IFIT-4) (Retinoic acid-induced gene G protein) (P60) (RIG-G)	IFIT3 CIG-49 IFI60 IFIT4 ISG60	Up-regulated
P00403	Cytochrome c oxidase subunit 2 (EC 7.1.1.9) (Cytochrome c oxidase polypeptide II)	MT-CO2 COII COX2 COXII MTCO2	Up-regulated
P19367	Hexokinase-1 (EC 2.7.1.1) (Brain form hexokinase) (Hexokinase type I) (HK I) (Hexokinase-A)	HK1	Up-regulated
P09110	3-ketoacyl-CoA thiolase, peroxisomal (EC 2.3.1.16) (Acetyl-CoA acyltransferase) (Beta-ketothiolase) (Peroxisomal 3-oxoacyl-CoA thiolase)	ACAA1 ACAA PTHIO	Up-regulated
O95497	Pantetheinase (EC 3.5.1.92) (Pantetheine hydrolase) (Tiff66) (Vascular non-inflammatory molecule 1) (Vanin-1)	VNN1	Up-regulated
Q9UBI6	Guanine nucleotide-binding protein G(I)/G(S)/G(O) subunit gamma-12	GNG12	Up-regulated

P12235	ADP/ATP translocase 1 (ADP,ATP carrier protein 1) (ADP,ATP carrier protein, heart/skeletal muscle isoform T1) (Adenine nucleotide translocator 1) (ANT 1) (Solute carrier family 25 member 4)	SLC25A4 ANT1	Up-regulated
P62805	Histone H4	H4C1 H4/A H4FA HIST1H4A; H4C2 H4/I H4FI HIST1H4B; H4C3 H4/G H4FG HIST1H4C; H4C4 H4/B H4FB HIST1H4D; H4C5 H4/J H4FJ HIST1H4E; H4C6 H4/C H4FC HIST1H4F; H4C8 H4/H H4FH HIST1H4H; H4C9 H4/M H4FM HIST1H4I; H4C11 H4/E H4FE HIST1H4J; H4C12 H4/D H4FD HIST1H4K; H4C13 H4/K H4FK HIST1H4L; H4C14 H4/N H4F2 H4FN HIST2H4 HIST2H4A; H4C15 H4/O H4FO HIST2H4B; H4-16 HIST4H4	Up-regulated
O95573	Long-chain-fatty-acid--CoA ligase 3 (EC 6.2.1.3) (Arachidonate--CoA ligase) (EC 6.2.1.15) (Long-chain acyl-CoA synthetase 3) (LACS 3)	ACSL3 ACS3 FACL3 LACS3	Up-regulated
O14737	Programmed cell death protein 5 (TF-1 cell apoptosis-related protein 19) (Protein TFAR19)	PDCD5 TFAR19	Up-regulated
P13010	X-ray repair cross-complementing protein 5 (EC 3.6.4.-) (86 kDa subunit of Ku antigen) (ATP-dependent DNA helicase 2 subunit 2) (ATP-dependent DNA helicase II 80 kDa subunit) (CTC box-binding factor 85 kDa subunit) (CTC85) (CTCBF) (DNA repair protein XRCC5) (Ku80) (Ku86) (Lupus Ku autoantigen protein p86) (Nuclear factor IV) (Thyroid-lupus autoantigen) (TLAA) (X-ray repair complementing defective repair in Chinese hamster cells 5 (double-strand-break rejoining))	XRCC5 G22P2	Up-regulated
P09913	Interferon-induced protein with tetratricopeptide repeats 2 (IFIT-2) (ISG-54 K) (Interferon-induced 54 kDa protein) (IFI-54K) (P54)	IFIT2 CIG-42 G10P2 IFI54 ISG54	Up-regulated
Q96Q42	Alsin (Amyotrophic lateral sclerosis 2 chromosomal region candidate gene 6 protein) (Amyotrophic lateral sclerosis 2 protein)	ALS2 ALS2CR6 KIAA1563	Down-regulated
P53618	Coatomer subunit beta (Beta-coat protein) (Beta-COP)	COPB1 COPB MSTP026	Down-regulated
O43294	Transforming growth factor beta-1-induced transcript 1 protein (Androgen receptor coactivator 55 kDa protein) (Androgen receptor-associated protein of 55 kDa)	TGFB1I1 ARA55	Down-regulated

	(Hydrogen peroxide-inducible clone 5 protein) (Hic-5)		
Q9NX63	MICOS complex subunit MIC19 (Coiled-coil-helix-coiled-coil-helix domain-containing protein 3)	CHCHD3 MIC19 MINOS3	Down-regulated
O43795	Unconventional myosin-Ib (MYH-1c) (Myosin I alpha) (MMI-alpha) (MMIa)	MYO1B	Down-regulated
Q9NP79	Vacuolar protein sorting-associated protein VTA1 homolog (Dopamine-responsive gene 1 protein) (DRG-1) (LYST-interacting protein 5) (LIP5) (SKD1-binding protein 1) (SBP1)	VTA1 C6orf55 HSPC228 My012	Down-regulated
Q9UNE7	E3 ubiquitin-protein ligase CHIP (EC 2.3.2.27) (Antigen NY-CO-7) (CLL-associated antigen KW-8) (Carboxy terminus of Hsp70-interacting protein) (RING-type E3 ubiquitin transferase CHIP) (STIP1 homology and U box-containing protein 1)	STUB1 CHIP PP1131	Down-regulated
Q9P2B4	CTTNBP2 N-terminal-like protein	CTTNBP2NL KIAA1433	Down-regulated
P46459	Vesicle-fusing ATPase (EC 3.6.4.6) (N-ethylmaleimide-sensitive fusion protein) (NEM-sensitive fusion protein) (Vesicular-fusion protein NSF)	NSF	Down-regulated
P21399	Cytoplasmic aconitate hydratase (Aconitase) (EC 4.2.1.3) (Citrate hydro-lyase) (Ferritin repressor protein) (Iron regulatory protein 1) (IRP1) (Iron-responsive element-binding protein 1) (IRE-BP 1)	ACO1 IREB1	Down-regulated
Q08211	ATP-dependent RNA helicase A (EC 3.6.4.13) (DEAH box protein 9) (DExH-box helicase 9) (Leukophysin) (LKP) (Nuclear DNA helicase II) (NDH II) (RNA helicase A)	DHX9 DDX9 LKP NDH2	Down-regulated
Q1KMD3	Heterogeneous nuclear ribonucleoprotein U-like protein 2 (Scaffold-attachment factor A2) (SAF-A2)	HNRNPUL2 HNRPUL2	Down-regulated
P54578	Ubiquitin carboxyl-terminal hydrolase 14 (EC 3.4.19.12) (Deubiquitinating enzyme 14) (Ubiquitin thioesterase 14) (Ubiquitin-specific-processing protease 14)	USP14 TGT	Down-regulated
Q04637	Eukaryotic translation initiation factor 4 gamma 1 (eIF-4-gamma 1) (eIF-4G 1) (eIF-4G1) (p220)	EIF4G1 EIF4F EIF4G EIF4GI	Down-regulated
Q15165	Serum paraoxonase/arylesterase 2 (PON 2) (EC 3.1.1.2) (EC 3.1.1.81) (Aromatic esterase 2) (A-esterase 2) (Serum aryldialkylphosphatase 2)	PON2	Down-regulated
P43487	Ran-specific GTPase-activating protein (Ran-binding protein 1) (RanBP1)	RANBP1	Down-regulated

P22061	Protein-L-isoaspartate(D-aspartate) O-methyltransferase (PIMT) (EC 2.1.1.77) (L-isoaspartyl protein carboxyl methyltransferase) (Protein L-isoaspartyl/D-aspartyl methyltransferase) (Protein-beta-aspartate methyltransferase)	PCMT1	Down-regulated
P16035	Metalloproteinase inhibitor 2 (CSC-21K) (Tissue inhibitor of metalloproteinases 2) (TIMP-2)	TIMP2	Down-regulated
O60884	DnaJ homolog subfamily A member 2 (Cell cycle progression restoration gene 3 protein) (Dnj3) (Dj3) (HIRA-interacting protein 4) (Renal carcinoma antigen NY-REN-14)	DNAJA2 CPR3 HIRIP4	Down-regulated
Q9Y333	U6 snRNA-associated Sm-like protein LSm2 (Protein G7b) (Small nuclear ribonuclear protein D homolog) (snRNP core Sm-like protein Sm-x5)	LSM2 C6orf28 G7B	Down-regulated
P55263	Adenosine kinase (AK) (EC 2.7.1.20) (Adenosine 5'-phosphotransferase)	ADK	Down-regulated
Q12792	Twinfilin-1 (Protein A6) (Protein tyrosine kinase 9)	TWF1 PTK9	Down-regulated
P83731	60S ribosomal protein L24 (60S ribosomal protein L30) (Large ribosomal subunit protein eL24)	RPL24	Down-regulated
O00571	ATP-dependent RNA helicase DDX3X (EC 3.6.4.13) (CAP-Rf) (DEAD box protein 3, X-chromosomal) (DEAD box, X isoform) (DBX) (Helicase-like protein 2) (HLP2)	DDX3X DBX DDX3	Down-regulated
P43243	Matrin-3	MATR3 KIAA0723	Down-regulated
O00629	Importin subunit alpha-3 (Importin alpha Q1) (Qip1) (Karyopherin subunit alpha-4)	KPNA4 QIP1	Down-regulated
Q9NTK5	Obg-like ATPase 1 (DNA damage-regulated overexpressed in cancer 45) (DOC45) (GTP-binding protein 9)	OLA1 GTPBP9 PRO2455 PTD004	Down-regulated
Q02809	Procollagen-lysine,2-oxoglutarate 5-dioxygenase 1 (EC 1.14.11.4) (Lysyl hydroxylase 1) (LH1)	PLOD1 LLH PLOD	Down-regulated
Q9NY33	Dipeptidyl peptidase 3 (EC 3.4.14.4) (Dipeptidyl aminopeptidase III) (Dipeptidyl arylamidase III) (Dipeptidyl peptidase III) (DPP III) (Enkephalinase B)	DPP3	Down-regulated
Q14194	Dihydropyrimidinase-related protein 1 (DRP-1) (Collapsin response mediator protein 1) (CRMP-1) (Inactive dihydropyrimidinase) (Unc-33-like phosphoprotein 3) (ULIP-3)	CRMP1 DPYSL1 ULIP3	Down-regulated
Q92896	Golgi apparatus protein 1 (CFR-1) (Cysteine-rich fibroblast growth factor receptor) (E-selectin ligand 1)	GLG1 CFR1 ESL1 MG160	Down-regulated

	(ESL-1) (Golgi sialoglycoprotein MG-160)		
P61313	60S ribosomal protein L15 (Large ribosomal subunit protein eL15)	RPL15 EC45 TCBAP0781	Down-regulated
Q9H0U4	Ras-related protein Rab-1B	RAB1B	Down-regulated
Q9NYF8	Bcl-2-associated transcription factor 1 (Btf) (BCLAF1 and THRAP3 family member 1)	BCLAF1 BTF KIAA0164	Down-regulated
Q9UNM6	26S proteasome non-ATPase regulatory subunit 13 (26S proteasome regulatory subunit RPN9) (26S proteasome regulatory subunit S11) (26S proteasome regulatory subunit p40.5)	PSMD13	Down-regulated
Q5JPE7	Nodal modulator 2 (pM5 protein 2)	NOMO2	Down-regulated
Q92890	Ubiquitin recognition factor in ER-associated degradation protein 1 (Ubiquitin fusion degradation protein 1) (UB fusion protein 1)	UFD1 UFD1L	Down-regulated
Q9Y2S6	Translation machinery-associated protein 7 (Coiled-coil domain-containing protein 72)	TMA7 CCDC72 HSPC016 HSPC330	Down-regulated
Q13510	Acid ceramidase (AC) (ACDase) (Acid CDase) (EC 3.5.1.23) (Acylsphingosine deacylase) (N-acylethanolamine hydrolase ASAH1) (EC 3.5.1.-) (N-acylsphingosine amidohydrolase) (Putative 32 kDa heart protein) (PHP32) [Cleaved into: Acid ceramidase subunit alpha; Acid ceramidase subunit beta]	ASAH1 ASAH HSD-33 HSD33	Down-regulated
P14927	Cytochrome b-c1 complex subunit 7 (Complex III subunit 7) (Complex III subunit VII) (QP-C) (Ubiquinol-cytochrome c reductase complex 14 kDa protein)	UQCRB UQBP	Down-regulated
P50570	Dynamin-2 (EC 3.6.5.5)	DNM2 DYN2	Down-regulated
Q7Z6Z7	E3 ubiquitin-protein ligase HUWE1 (EC 2.3.2.26) (ARF-binding protein 1) (ARF-BP1) (HECT, UBA and WWE domain-containing protein 1) (HECT-type E3 ubiquitin transferase HUWE1) (Homologous to E6AP carboxyl terminus homologous protein 9) (HectH9) (Large structure of UREB1) (LASU1) (Mcl-1 ubiquitin ligase E3) (Mule) (Upstream regulatory element-binding protein 1) (URE-B1) (URE-binding protein 1)	HUWE1 KIAA0312 KIAA1578 UREB1 HSPC272	Down-regulated
Q92598	Heat shock protein 105 kDa (Antigen NY-CO-25) (Heat shock 110 kDa protein)	HSPH1 HSP105 HSP110 KIAA0201	Down-regulated
O00244	Copper transport protein ATOX1 (Metal transport protein ATX1)	ATOX1 HAH1	Down-regulated

O00231	26S proteasome non-ATPase regulatory subunit 11 (26S proteasome regulatory subunit RPN6) (26S proteasome regulatory subunit S9) (26S proteasome regulatory subunit p44.5)	PSMD11	Down-regulated
Q8WUP2	Filamin-binding LIM protein 1 (FBLP-1) (Migfilin) (Mitogen-inducible 2-interacting protein) (MIG2-interacting protein)	FBLIM1 FBLP1	Down-regulated
P25786	Proteasome subunit alpha type-1 (EC 3.4.25.1) (30 kDa prosomal protein) (PROS-30) (Macropain subunit C2) (Multicatalytic endopeptidase complex subunit C2) (Proteasome component C2) (Proteasome nu chain)	PSMA1 HC2 NU PROS30 PSC2	Down-regulated
P05121	Plasminogen activator inhibitor 1 (PAI) (PAI-1) (Endothelial plasminogen activator inhibitor) (Serpin E1)	SERPINE1 PAI1 PLANH1	Down-regulated
P52597	Heterogeneous nuclear ribonucleoprotein F (hnRNP F) (Nucleolin-like protein mcs94-1) [Cleaved into: Heterogeneous nuclear ribonucleoprotein F, N-terminally processed]	HNRNPF HNRPF	Down-regulated
Q9UBR2	Cathepsin Z (EC 3.4.18.1) (Cathepsin P) (Cathepsin X)	CTSZ	Down-regulated
O43615	Mitochondrial import inner membrane translocase subunit TIM44	TIMM44 MIMT44 TIM44	Down-regulated
P60468	Protein transport protein Sec61 subunit beta	SEC61B	Down-regulated
P61513	60S ribosomal protein L37a (Large ribosomal subunit protein eL43)	RPL37A	Down-regulated
P10253	Lysosomal alpha-glucosidase (EC 3.2.1.20) (Acid maltase) (Aglucosidase alfa) [Cleaved into: 76 kDa lysosomal alpha-glucosidase; 70 kDa lysosomal alpha-glucosidase]	GAA	Down-regulated
P42766	60S ribosomal protein L35 (Large ribosomal subunit protein uL29)	RPL35	Down-regulated
P12270	Nucleoprotein TPR (Megator) (NPC-associated intranuclear protein) (Translocated promoter region protein)	TPR	Down-regulated
Q15404	Ras suppressor protein 1 (RSP-1) (Rsu-1)	RSU1 RSP1	Down-regulated
P05198	Eukaryotic translation initiation factor 2 subunit 1 (Eukaryotic translation initiation factor 2 subunit alpha) (eIF-2-alpha) (eIF-2A) (eIF-2alpha)	EIF2S1 EIF2A	Down-regulated
P48047	ATP synthase subunit O, mitochondrial (ATP synthase peripheral stalk subunit OSCP) (Oligomycin sensitivity conferral protein) (OSCP)	ATP5PO ATP5O ATPO	Down-regulated

Q15121	Astrocytic phosphoprotein PEA-15 (15 kDa phosphoprotein enriched in astrocytes) (Phosphoprotein enriched in diabetes) (PED)	PEA15	Down-regulated
Q14677	Clathrin interactor 1 (Clathrin-interacting protein localized in the trans-Golgi region) (Clint) (Enthoprotin) (Epsin-4) (Epsin-related protein) (EpsinR)	CLINT1 ENTH EPN4 EPNR KIAA0171	Down-regulated
Q99426	Tubulin-folding cofactor B (Cytoskeleton-associated protein 1) (Cytoskeleton-associated protein CKAPI) (Tubulin-specific chaperone B)	TBCB CG22 CKAP1	Down-regulated
Q9UHB6	LIM domain and actin-binding protein 1 (Epithelial protein lost in neoplasm)	LIMA1 EPLIN SREBP3 PP624	Down-regulated
Q562R1	Beta-actin-like protein 2 (Kappa-actin)	ACTBL2	Down-regulated
P83916	Chromobox protein homolog 1 (HP1Hsbeta) (Heterochromatin protein 1 homolog beta) (HP1 beta) (Heterochromatin protein p25) (M31) (Modifier 1 protein) (p25beta)	CBX1 CBX	Down-regulated
P52943	Cysteine-rich protein 2 (CRP-2) (Protein ESP1)	CRIP2 CRP2	Down-regulated
Q14192	Four and a half LIM domains protein 2 (FHL-2) (LIM domain protein DRAL) (Skeletal muscle LIM-protein 3) (SLIM-3)	FHL2 DRAL SLIM3	Down-regulated
Q9UKY7	Protein CDV3 homolog	CDV3 H41	Down-regulated
Q13409	Cytoplasmic dynein 1 intermediate chain 2 (Cytoplasmic dynein intermediate chain 2) (Dynein intermediate chain 2, cytosolic) (DH IC-2)	DYNC1I2 DNCI2 DNCIC2	Down-regulated
P26599	Polypyrimidine tract-binding protein 1 (PTB) (57 kDa RNA-binding protein PPTB-1) (Heterogeneous nuclear ribonucleoprotein I) (hnRNP I)	PTBP1 PTB	Down-regulated
O60749	Sorting nexin-2 (Transformation-related gene 9 protein) (TRG-9)	SNX2 TRG9	Down-regulated

Table S3. Full list of differentially expressed proteins in tip vs. pal, p < 0.01 and FDR < 0.1.

Entry	Protein names	Gene names	Protein expression
O94905	Erlin-2 (Endoplasmic reticulum lipid raft-associated protein 2) (Stomatin-prohibitin-flotillin-HflC/K domain-containing protein 2) (SPFH domain-containing protein 2)	ERLIN2 C8orf2 SPFH2 UNQ2441/PRO5003/PRO9924	Up-regulated
P62829	60S ribosomal protein L23 (60S ribosomal protein L17) (Large ribosomal subunit protein uL14)	RPL23	Up-regulated
Q6NYC8	Phostensin (Protein phosphatase 1 F-actin cytoskeleton-targeting subunit) (Protein phosphatase 1 regulatory subunit 18)	PPP1R18 HKMT1098 KIAA1949	Up-regulated
O75340	Programmed cell death protein 6 (Apoptosis-linked gene 2 protein homolog) (ALG-2)	PDCD6 ALG2	Up-regulated
P20290	Transcription factor BTF3 (Nascent polypeptide-associated complex subunit beta) (NAC-beta) (RNA polymerase B transcription factor 3)	BTF3 NACB OK/SW-cl.8	Up-regulated
Q9UK76	Jupiter microtubule associated homolog 1 (Androgen-regulated protein 2) (Hematological and neurological expressed 1 protein) [Cleaved into: Jupiter microtubule associated homolog 1, N-terminally processed]	JPT1 ARM2 HN1	Up-regulated
P53992	Protein transport protein Sec24C (SEC24-related protein C)	SEC24C KIAA0079	Up-regulated
P07305	Histone H1.0 (Histone H1') (Histone H1(0)) [Cleaved into: Histone H1.0, N-terminally processed]	H1-0 H1F0 H1FV	Up-regulated
Q92882	Osteoclast-stimulating factor 1	OSTF1	Up-regulated
P28062	Proteasome subunit beta type-8 (EC 3.4.25.1) (Low molecular mass protein 7) (Macropain subunit C13) (Multicatalytic endopeptidase complex subunit C13) (Proteasome component C13) (Proteasome subunit beta-5i) (Really interesting new gene 10 protein)	PSMB8 LMP7 PSMB5i RING10 Y2	Up-regulated
P27708	CAD protein [Includes: Glutamine-dependent carbamoyl-phosphate synthase (EC 6.3.5.5); Aspartate carbamoyltransferase (EC 2.1.3.2); Dihydroorotase (EC 3.5.2.3)]	CAD	Up-regulated
Q13561	Dynactin subunit 2 (50 kDa dynein-associated polypeptide) (Dynactin complex 50 kDa subunit) (DCTN-50) (p50 dynamitin)	DCTN2 DCTN50	Up-regulated

Q9UHB9	Signal recognition particle subunit SRP68 (SRP68) (Signal recognition particle 68 kDa protein)	SRP68	Up-regulated
Q99729	Heterogeneous nuclear ribonucleoprotein A/B (hnRNP A/B) (APOBEC1-binding protein 1) (ABBP-1)	HNRNPAB ABBP1 HNRPAB	Up-regulated
Q16204	Coiled-coil domain-containing protein 6 (Papillary thyroid carcinoma-encoded protein) (Protein H4)	CCDC6 D10S170 TST1	Up-regulated
P78344	Eukaryotic translation initiation factor 4 gamma 2 (eIF-4-gamma 2) (eIF-4G 2) (eIF4G 2) (Death-associated protein 5) (DAP-5) (p97)	EIF4G2 DAP5 OK/SW-cl.75	Up-regulated
Q02750	Dual specificity mitogen-activated protein kinase kinase 1 (MAP kinase kinase 1) (MAPKK 1) (MKK1) (EC 2.7.12.2) (ERK activator kinase 1) (MAPK/ERK kinase 1) (MEK 1)	MAP2K1 MEK1 PRKMK1	Up-regulated
P12955	Xaa-Pro dipeptidase (X-Pro dipeptidase) (EC 3.4.13.9) (Imidodipeptidase) (Peptidase D) (Proline dipeptidase) (Prolidase)	PEPD PRD	Down-regulated
Q9NX63	MICOS complex subunit MIC19 (Coiled-coil-helix-coiled-coil-helix domain-containing protein 3)	CHCHD3 MIC19 MINOS3	Down-regulated
Q15165	Serum paraoxonase/arylesterase 2 (PON 2) (EC 3.1.1.2) (EC 3.1.1.81) (Aromatic esterase 2) (A-esterase 2) (Serum aryldialkylphosphatase 2)	PON2	Down-regulated
Q9UJU6	Drebrin-like protein (Cervical SH3P7) (Cervical mucin-associated protein) (Drebrin-F) (HPK1-interacting protein of 55 kDa) (HIP-55) (SH3 domain-containing protein 7)	DBNL CMAP SH3P7 PP5423	Down-regulated
O43294	Transforming growth factor beta-1-induced transcript 1 protein (Androgen receptor coactivator 55 kDa protein) (Androgen receptor-associated protein of 55 kDa) (Hydrogen peroxide-inducible clone 5 protein) (Hic-5)	TGFB1I1 ARA55	Down-regulated
P21399	Cytoplasmic aconitate hydratase (Aconitase) (EC 4.2.1.3) (Citrate hydro-lyase) (Ferritin repressor protein) (Iron regulatory protein 1) (IRP1) (Iron-responsive element-binding protein 1) (IRE-BP 1)	ACO1 IREB1	Down-regulated
Q9NZ01	Very-long-chain enoyl-CoA reductase (EC 1.3.1.93) (Synaptic glycoprotein SC2) (Trans-2,3-enoyl-CoA reductase) (TER)	TECR GPSN2 SC2	Down-regulated
P53618	Coatomer subunit beta (Beta-coat protein) (Beta-COP)	COPB1 COPB MSTP026	Down-regulated

O43615	Mitochondrial import inner membrane translocase subunit TIM44	TIMM44 MIMT44 TIM44	Down-regulated
Q12792	Twinfilin-1 (Protein A6) (Protein tyrosine kinase 9)	TWF1 PTK9	Down-regulated
P14927	Cytochrome b-c1 complex subunit 7 (Complex III subunit 7) (Complex III subunit VII) (QP-C) (Ubiquinol-cytochrome c reductase complex 14 kDa protein)	UQCRB UQBP	Down-regulated
Q9NY33	Dipeptidyl peptidase 3 (EC 3.4.14.4) (Dipeptidyl aminopeptidase III) (Dipeptidyl arylamidase III) (Dipeptidyl peptidase III) (DPP III) (Enkephalinase B)	DPP3	Down-regulated
P54578	Ubiquitin carboxyl-terminal hydrolase 14 (EC 3.4.19.12) (Deubiquitinating enzyme 14) (Ubiquitin thioesterase 14) (Ubiquitin-specific-processing protease 14)	USP14 TGT	Down-regulated
Q9NTK5	Obg-like ATPase 1 (DNA damage-regulated overexpressed in cancer 45) (DOC45) (GTP-binding protein 9)	OLA1 GTPBP9 PRO2455 PTD004	Down-regulated
Q9H0U4	Ras-related protein Rab-1B	RAB1B	Down-regulated
P42167	Lamina-associated polypeptide 2, isoforms beta/gamma (Thymopoietin, isoforms beta/gamma) (TP beta/gamma) (Thymopoietin-related peptide isoforms beta/gamma) (TPRP isoforms beta/gamma) [Cleaved into: Thymopoietin (TP) (Splenin); Thymopentin (TP5)]	TMPO LAP2	Down-regulated
P10253	Lysosomal alpha-glucosidase (EC 3.2.1.20) (Acid maltase) (Aglucosidase alfa) [Cleaved into: 76 kDa lysosomal alpha-glucosidase; 70 kDa lysosomal alpha-glucosidase]	GAA	Down-regulated
O95336	6-phosphogluconolactonase (6PGL) (EC 3.1.1.31)	PGLS	Down-regulated
P52597	Heterogeneous nuclear ribonucleoprotein F (hnRNP F) (Nucleolin-like protein mcs94-1) [Cleaved into: Heterogeneous nuclear ribonucleoprotein F, N-terminally processed]	HNRNPF HNRPF	Down-regulated
O76094	Signal recognition particle subunit SRP72 (SRP72) (Signal recognition particle 72 kDa protein)	SRP72	Down-regulated
Q5JPE7	Nodal modulator 2 (pM5 protein 2)	NOMO2	Down-regulated
P83916	Chromobox protein homolog 1 (HP1Hsbeta) (Heterochromatin protein 1 homolog beta) (HP1	CBX1 CBX	Down-regulated

	beta) (Heterochromatin protein p25) (M31) (Modifier 1 protein) (p25beta)		
O00231	26S proteasome non-ATPase regulatory subunit 11 (26S proteasome regulatory subunit RPN6) (26S proteasome regulatory subunit S9) (26S proteasome regulatory subunit p44.5)	PSMD11	Down-regulated
Q15046	Lysine--tRNA ligase (EC 2.7.7.-) (EC 6.1.1.6) (Lysyl-tRNA synthetase) (LysRS)	KARS1 KARS KIAA0070	Down-regulated
O43795	Unconventional myosin-Ib (MYH-1c) (Myosin I alpha) (MMI-alpha) (MMIa)	MYO1B	Down-regulated
Q9BZL1	Ubiquitin-like protein 5	UBL5	Down-regulated
Q16775	Hydroxyacylglutathione hydrolase, mitochondrial (EC 3.1.2.6) (Glyoxalase II) (Glx II)	HAGH GLO2 HAGH1	Down-regulated
P52943	Cysteine-rich protein 2 (CRP-2) (Protein ESP1)	CRIP2 CRP2	Down-regulated

Table S4. Full list of proteins that were unique in pal vs. veh, $p < 0.01$ and FDR < 0.1.

Entry	Protein names	Gene names	Protein expression
P31949	Protein S100-A11 (Calgizzarin) (Metastatic lymph node gene 70 protein) (MLN 70) (Protein S100-C) (S100 calcium-binding protein A11) [Cleaved into: Protein S100-A11, N-terminally processed]	S100A11 MLN70 S100C	Up-regulated
Q9P2R7	Succinate--CoA ligase [ADP-forming] subunit beta, mitochondrial (EC 6.2.1.5) (ATP-specific succinyl-CoA synthetase subunit beta) (A-SCS) (Succinyl-CoA synthetase beta-A chain) (SCS-betaA)	SUCLA2	Up-regulated
Q9Y265	RuvB-like 1 (EC 3.6.4.12) (49 kDa TATA box-binding protein-interacting protein) (49 kDa TBP-interacting protein) (54 kDa erythrocyte cytosolic protein) (ECP-54) (INO80 complex subunit H) (Nuclear matrix protein 238) (NMP 238) (Pontin 52) (TIP49a) (TIP60-associated protein 54-alpha) (TAP54-alpha)	RUVBL1 INO80H NMP238 TIP49 TIP49A	Up-regulated
P61204	ADP-ribosylation factor 3	ARF3	Up-regulated
Q8NGA1	Olfactory receptor 1M1 (Olfactory receptor 19-6) (OR19-6) (Olfactory receptor OR19-5)	OR1M1	Up-regulated
P81605	Dermcidin (EC 3.4.-.-) (Preproteolysin) [Cleaved into: Survival-promoting peptide; DCD-1]	DCD AIDD DSEP	Up-regulated
P13473	Lysosome-associated membrane glycoprotein 2 (LAMP-2) (Lysosome-associated membrane protein 2) (CD107 antigen-like family member B) (LGP-96) (CD antigen CD107b)	LAMP2	Up-regulated
Q6P2Q9	Pre-mRNA-processing-splicing factor 8 (220 kDa U5 snRNP-specific protein) (PRP8 homolog) (Splicing factor Prp8) (p220)	PRPF8 PRPC8	Up-regulated
P46109	Crk-like protein	CRKL	Up-regulated
P49821	NADH dehydrogenase [ubiquinone] flavoprotein 1, mitochondrial (EC 7.1.1.2) (Complex I-51kD) (CI-51kD) (NADH dehydrogenase flavoprotein 1) (NADH-ubiquinone oxidoreductase 51 kDa subunit)	NDUFV1 UQOR1	Up-regulated
Q9NZ01	Very-long-chain enoyl-CoA reductase (EC 1.3.1.93) (Synaptic glycoprotein SC2) (Trans-2,3-enoyl-CoA reductase) (TER)	TECR GPSN2 SC2	Up-regulated
Q9UJU6	Drebrin-like protein (Cervical SH3P7) (Cervical mucin-associated protein) (Drebrin-F) (HPK1-interacting protein of 55 kDa) (HIP-55) (SH3 domain-containing protein 7)	DBNL CMAP SH3P7 PP5423	Up-regulated
P12955	Xaa-Pro dipeptidase (X-Pro dipeptidase) (EC 3.4.13.9) (Imidodipeptidase) (Peptidase D) (Proline dipeptidase) (Prolidase)	PEPD PRD	Up-regulated
P78344	Eukaryotic translation initiation factor 4 gamma 2 (eIF-4-gamma 2) (eIF-4G 2) (eIF4G 2) (Death-associated protein 5) (DAP-5) (p97)	EIF4G2 DAP5 OK/SW-cl.75	Down-regulated
Q16204	Coiled-coil domain-containing protein 6 (Papillary thyroid carcinoma-encoded protein) (Protein H4)	CCDC6 D10S170 TST1	Down-regulated
Q13561	Dynactin subunit 2 (50 kDa dynein-associated polypeptide) (Dynactin complex 50 kDa subunit) (DCTN-50) (p50 dynamitin)	DCTN2 DCTN50	Down-regulated
Q9NPA8	Transcription and mRNA export factor ENY2 (Enhancer of yellow 2 transcription factor homolog)	ENY2 DC6	Down-regulated
P61927	60S ribosomal protein L37 (G1.16) (Large ribosomal subunit protein eL37)	RPL37	Down-regulated

O95373	Importin-7 (Imp7) (Ran-binding protein 7) (RanBP7)	IPO7 RANBP7	Down-regulated
P84090	Enhancer of rudimentary homolog	ERH	Down-regulated
Q6NYC8	Phostensin (Protein phosphatase 1 F-actin cytoskeleton-targeting subunit) (Protein phosphatase 1 regulatory subunit 18)	PPP1R18 HKMT1098 KIAA1949	Down-regulated
Q9UHV9	Prefoldin subunit 2	PFDN2 PFD2 HSPC231	Down-regulated
P53621	Coatomer subunit alpha (Alpha-coat protein) (Alpha-COP) (HEP-COP) (HEPCOP) [Cleaved into: Xenin (Xenopsin-related peptide); Proxenin]	COPA	Down-regulated

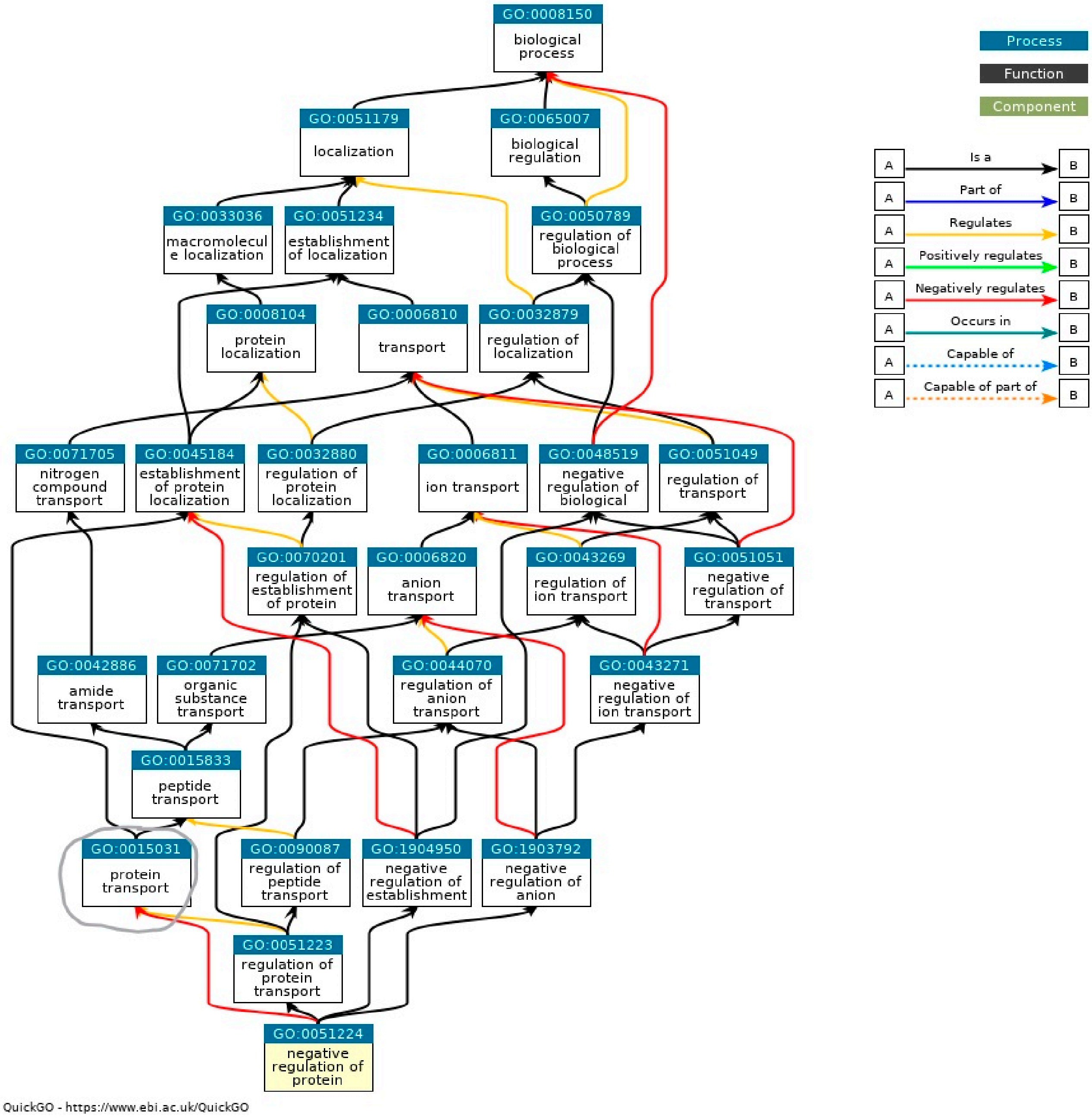

Figure S2. GO Slim of the regulation of protein transpor

Table S5. Full list of nodes that passed the filter imposed by the MCODE algorithm in Cytoscape 3.8.0, representing the hub proteins obtained in this study, positively and significantly correlated to changes induced by treatment.

Mcode Cluster	From node	Gene names	Belonging module	Node name	To node
12	O75340	PDCD6 ALG2	turquoise	O75340	O75340
12	P51571	SSR4 TRAPD	turquoise	P51571	P51571
12	P13861	PRKAR2A PKR2 PRKAR2	turquoise	P13861	
11	Q9NY33	DPP3	turquoise	Q9NY33	Q9NY33
11	O43294	TGFB1I1 ARA55	turquoise	O43294	
13		PLCH1 KIAA1069 PLCL3	red	Q4KWH8	Q4KWH8
13	Q9BZK7	TBL1XR1 IRA1 TBLR1	red	Q9BZK7	Q9BZK7
13	P49023	PXN	red	P49023	
4		ALS2 ALS2CR6 KIAA1563	red	Q96Q42	Q96Q42
6	P55263	ADK	red	P55263	P55263
6	O95373	IPO7 RANBP7	red	O95373	O95373
14	P61313	RPL15 EC45 TCBAP0781	red	P61313	P61313
14	P47755	CAPZA2	red	P47755	
4	O60884	DNAJA2 CPR3 HIRIP4	red	O60884	O60884
5		CHCHD3 MIC19 MINOS3	turquoise	Q9NX63	Q9NX63
5	P10253	GAA	turquoise	P10253	P10253
11	Q9NTK5	OLA1 GTPBP9 PRO2455 PTD004	turquoise	Q9NTK5	Q9NTK5
8		EIF3L EIF3EIP EIF3S6IP HSPC021 HSPC025 MSTP005	turquoise	Q9Y262	Q9Y262
8	P84157	MXRA7	turquoise	P84157	P84157
8	P13611	VCAN CSPG2	turquoise	P13611	P13611
8	P09960	LTA4H LTA4	turquoise	P09960	
4	P16035	TIMP2	red	P16035	P16035

6	P61204	ARF3	red	P61204	P61204
14	O43795	MYO1B	red	O43795	O43795
5	Q12792	TWF1 PTK9	turquoise	Q12792	Q12792
5	P21399	ACO1 IREB1	turquoise	P21399	P21399
4	P60484	PTEN MMAC1 TEP1	red	P60484	P60484
6	Q92890	UFD1 UFD1L	red	Q92890	Q92890
6		STUB1 CHIP PP1131	red	Q9UNE7	Q9UNE7
2	P63173	RPL38	pink	P63173	P63173
2	Q9BR76	CORO1B	pink	Q9BR76	Q9BR76
4	O00571	DDX3X DBX DDX3	red	O00571	O00571
2	P08708	RPS17 RPS17L	pink	P08708	P08708
2	P37837	TALDO1 TAL TALDO TALDOR	pink	P37837	P37837
2	P49773	HINT1 HINT PKCI1 PRKCNH1	pink	P49773	P49773
2	P23396	RPS3 OK/SW-cl.26	pink	P23396	P23396
2	P23381	WARS1 IFI53 WARS WRS	pink	P23381	P23381
2	P30086	PEBP1 PBP PEBP	pink	P30086	P30086
2	P07951	TPM2 TMSB	pink	P07951	P07951
2	P26038	MSN	pink	P26038	P26038
2	P13639	EEF2 EF2	pink	P13639	P13639
1		ART4 DO DOK1	turquoise	Q93070	Q93070
1	O95819	MAP4K4 HGK KIAA0687 NIK	turquoise	O95819	O95819
10		PFDN1 PFD1	turquoise	O60925	O60925
1	P49902	NT5C2 NT5B NT5CP PNT5	turquoise	P49902	P49902
1	P18065	IGFBP2 BP2 IBP2	turquoise	P18065	P18065
1	Q7L014	DDX46 KIAA0801	turquoise	Q7L014	Q7L014
7	P36507	MAP2K2 MEK2 MKK2 PRKMK2	pink	P36507	P36507
3	Q9Y646	CPQ LCH1 PGCP	pink	Q9Y646	Q9Y646
1	O95497	VNN1	turquoise	O95497	O95497

7	Q6IBS0	TWF2 PTK9L MSTP011	turquoise	Q6IBS0	Q6IBS0
1	P05413	FABP3 FABP11 MDGI	turquoise	P05413	P05413
1	P62244	RPS15A OK/SW-cl.82	turquoise	P62244	P62244
1	Q9P0K7	RAI14 KIAA1334 NORPEG	turquoise	Q9P0K7	Q9P0K7
1	Q9NVD7	PARVA MXRA2	turquoise	Q9NVD7	Q9NVD7
1	P53985	SLC16A1 MCT1	turquoise	P53985	P53985
1	P32969	RPL9 OK/SW-cl.103; RPL9P7; RPL9P8; RPL9P9	turquoise	P32969	P32969
7	P05121	SERPINE1 PAI1 PLANH1	red	P05121	P05121
1	P30040	ERP29 C12orf8 ERP28	turquoise	P30040	P30040
1	P49327	FASN FAS	turquoise	P49327	P49327
1	O00231	PSMD11	turquoise	O00231	O00231
1	P39687	ANP32A C15orf1 LANP MAPM PHAP1	turquoise	P39687	P39687
1	B5ME19	EIF3CL	turquoise	B5ME19	B5ME19
2	P27635	RPL10 DXS648E QM	turquoise	P27635	P27635
10	Q07666	KHDRBS1 SAM68	turquoise	Q07666	Q07666
2	P49257	LMAN1 ERGIC53 F5F8D	turquoise	P49257	P49257
1	P62195	PSMC5 SUG1	turquoise	P62195	P62195
2	O00159	MYO1C	turquoise	O00159	O00159
1	P12235	SLC25A4 ANT1	turquoise	P12235	P12235
1	P24821	TNC HXB	turquoise	P24821	P24821
1	P11766	ADH5 ADHX FDH	turquoise	P11766	P11766
7	Q04637	EIF4G1 EIF4F EIF4G EIF4GI	turquoise	Q04637	Q04637
1	Q92499	DDX1	turquoise	Q92499	Q92499
10	P15559	NQO1 DIA4 NMOR1	turquoise	P15559	P15559

1	O14974	PPP1R12A MBS MYPT1	turquoise	O14974	O14974
1	P07737	PFN1	turquoise	P07737	P07737
2	Q16527	CSRP2 LMO5 SMLIM	turquoise	Q16527	Q16527
1	Q14247	CTTN EMS1	turquoise	Q14247	Q14247
1	P29692	EEF1D EF1D	turquoise	P29692	P29692
7	Q9Y617	PSAT1 PSA	pink	Q9Y617	Q9Y617
9	Q00341	HDLBP HBP VGL	turquoise	Q00341	Q00341
9	P14136	GFAP	turquoise	P14136	P14136
2	Q99497	PARK7	turquoise	Q99497	Q99497
1	P27348	YWHAQ	turquoise	P27348	P27348
1	P23528	CFL1 CFL	turquoise	P23528	P23528
1	Q06830	PRDX1 PAGA PAGB TDPX2	turquoise	Q06830	Q06830
1	P62258	YWHAE	turquoise	P62258	P62258
3	P50395	GDI2 RABGDIB	turquoise	P50395	P50395
1	P07195	LDHB	turquoise	P07195	P07195
3	P13667	PDIA4 ERP70 ERP72	turquoise	P13667	P13667
1	P55072	VCP	turquoise	P55072	P55072
1	O75083	WDR1	turquoise	O75083	O75083
1	P68104	EEF1A1 EEF1A EF1A LENG7	turquoise	P68104	P68104
9	P63104	YWHAZ	turquoise	P63104	P63104
3	P30101	PDIA3 ERP57 ERP60 GRP58	turquoise	P30101	P30101
2	Q14195	DPYSL3 CRMP4 DRP3 ULIP ULIP1	turquoise	Q14195	Q14195
1	Q13509	TUBB3 TUBB4	turquoise	Q13509	Q13509
1	P07900	HSP90AA1 HSP90A HSPC1 HSPCA	turquoise	P07900	P07900
7	P14625	HSP90B1 GRP94 TRA1	turquoise	P14625	P14625
1	P07437	TUBB TUBB5 OK/SW-cl.56	turquoise	P07437	P07437
1	Q71U36	TUBA1A TUBA3	turquoise	Q71U36	Q71U36
1	P63261	ACTG1 ACTG	turquoise	P63261	

3	P09913	IFIT2 CIG-42 G10P2 IFI54 ISG54	turquoise	P09913	P09913
7	Q9UBI6	GNG12	turquoise	Q9UBI6	Q9UBI6
3	O14879	IFIT3 CIG-49 IFI60 IFIT4 ISG60	turquoise	O14879	O14879
1	P09211	GSTP1 FAEES3 GST3	turquoise	P09211	P09211
1	O14950	MYL12B MRLC2 MYLC2B	turquoise	O14950	O14950
7	P11021	HSPA5 GRP78	turquoise	P11021	P11021
3	P04406	GAPDH GAPD CDABP0047 OK/SW-cl.12	turquoise	P04406	P04406
1	P11142	HSPA8 HSC70 HSP73 HSPA10	turquoise	P11142	P11142
